# Vertically and horizontally transmitted microbial symbionts shape the gut microbiota ontogenesis of a skin-mucus feeding discus fish progeny

**DOI:** 10.1038/s41598-017-05662-w

**Published:** 2017-07-12

**Authors:** François-Étienne Sylvain, Nicolas Derome

**Affiliations:** Institut de biologie intégrative et des systèmes, Université Laval, Biology Department, 1030, avenue de la Médecine, Quebec (QC), Canada G1V 0A6

## Abstract

Fish gut microbial communities play key functions for their hosts, but their ontogenesis is poorly understood. Recent studies on the zebrafish suggest that gut symbionts are recruited naturally through horizontal transmission from environmental water. We used an alternative fish model, the discus (*Symphysodon aequifasciata*), to identify the main factors driving fish gut microbiota ontogenesis. The discus exhibits a unique parenting behavior: both discus parents vertically feed their fry with a cutaneous mucus secretion during three weeks post-hatching. We hypothesized that vertical microbial transmission via parental mucus feeding, along with horizontal transmission of environmental microbial symbionts, helps to shape the taxonomic structure of the discus fry gut microbiota. To assess this premise, we thoroughly documented the gut microbiota ontogenesis of a discus progeny during 100 days post-hatching. The V4 16S rRNA gene was sequenced to assess taxonomic structure of fry gut, parent mucus, and water samples. Our main results suggest that specific microbial symbionts both from the parents skin mucus and environmental water play important roles in shaping the structure of the fry gut microbiota.

## Introduction

The gut microbiota plays major roles in immunity^1^, growth^2^, and energy utilization^3^ of the host. Ontogenesis of mature gut microbial communities has been largely documented in mammals. Studies show that in human infants, gut microbiota colonization is mainly controlled by extrinsic factors such as the composition of the maternal skin microbiota, the mode of delivery, and most importantly by the first diet of the neonate (i.e. colostrum and subsequent mother’s milk)^4, 5^.

On teleost hosts, only few studies have investigated gut microbiota ontogenesis so far. This is a topic of interest, as the sequence of the initial microbial colonization of the gut has important implications in the yield of aquaculture activities, both in terms of fish growth rate^6^ and disease susceptibility^7^. Those parameters are critically dependent upon maturation of fish gut microbiome, which is controlled by the composition of water microbial community^7^ and the components of the first feed to the fry^8^. The general understanding is that fish gut microbiota symbionts are mainly horizontally recruited from the environment (i.e. surrounding water, food) (reviewed in ref. 7), where neutral processes such as drift and dispersal generate most of the microbial diversity^9^. Accordingly, Stephens *et al*.^10^ have shown that bacterial communities associated with zebrafish larvae (*Danio rerio*) exhibited low inter-individual variation and were more similar to the surrounding environmental communities than were adult fish microbiota, thus highlighting the role of environmental exposure in driving gut microbial recruitment at early developmental stages. At later stages, gut microbiota gradually differentiated from environmental community, and exhibited more inter-individual variation^10^. The same overall pattern was observed for wild Atlantic salmon populations, where environmental factors lost their potential to shape gut microbiota in later life stages^7^. Therefore, early gut microbiota ontogenesis in most fish appears to be different from mammalian gut microbial ontogenesis, in which breastfeeding allows the early vertical transmission of a specific gut pioneering community that differs from the environmental community^11^.

To investigate further the contribution of different factors in shaping fish gut microbiota, we thoroughly documented the gut microbiota ontogenesis of a progeny of discus fish (*Symphysodon aequifasciata*). The discus fish is a fascinating alternative fish model, as it displays an unusual mammalian-like parental care behavior: free-swimming discus fry feed exclusively on a specific cutaneous mucus secretion transmitted vertically from both parents during three weeks post-hatching^12^. This mucus feeding behavior (termed “contacting”) has evolved several times in fish lineages and is also observed in more than 28 other species, although the behavior in most species is usually less marked than in discus^13^. Some beneficial components of discus mucus that are vertically transmitted to the fry are analog to those of mammalian milk: the substance is rich in immune related proteins such as immunoglobulins and lectins, other proteins, hormones, ions, amino acids, and antibodies^12, 14–16^. Interestingly, many of these components (prolactin, immunoglobulins, and specific proteins) increase significantly in the skin mucus during breeding^12, 14–16^.

Skipper (1956)^17^ suggested that discus larvae were also feeding on microorganisms that lived commensally on their parent’s skin mucus layer. This suggestion has never been investigated further since 1956. With the development of high throughput sequencing technologies, it is now well known that bacterial symbionts are abundant within fish skin mucus^18^. Thus, as discus fry feed on the cutaneous mucus of their parents, they inevitably ingest parental skin mucus bacteria, which are therefore vertically transmitted from the parents to the progeny. Whether these parental symbionts settle in the discus fry gut or only go through it, is not known. We hypothesize that this vertical microbial transmission from the parental skin mucus, along with neutral horizontal transmission of environmental microbial symbionts, helps to shape the taxonomic structure of the discus fry gut microbiota.

To our knowledge, the ontogenesis of the gut microbiota of teleosts exhibiting specialized vertical feeding parental care behavior, such as *Symphysodon aequifasciata*, has never been documented. In our study, we aimed to: (1) describe the gut microbiota ontogenesis of a progeny of discus fry; (2) identify the contribution of the different microbial reservoirs (water, parents mucus, adult diet) in shaping the mature gut microbiota; and (3) document potential taxonomic shifts in the cutaneous mucus microbiota of adult discus during the parenting phase. To achieve these objectives, we documented the taxonomic structure of microbial communities associated with the gut, cutaneous mucus and surrounding water of one breeding discus pair and their fry along a 100 days post-hatching (DPH) experiment. We used high-throughput amplicon sequencing of the 16S rRNA gene to characterize the taxonomic structure of bacterial communities. In addition to the traditional approach used to describe gut microbiota ontogeny (e.g. measuring and characterizing microbial diversity through time), we used a novel network-based approach to identify the interactions between the most abundant microbial players driving the gut microbial colonization.

## Results

A total of 53 samples have been collected, including: 2 whole fry samples from 0–3 DPH, 5 whole fry samples from 4–20 DPH, 5 fry feces samples from 50–80 DPH, 4 fry feces samples from 80–100 DPH, 8 adult feces samples, 4 breeding discus mucus samples, 8 non-breeding discus mucus samples, 16 water samples, and 1 adult food sample. After amplification of the 16S rRNA V4 region, all libraries were sequenced on the MiSeq platform (Illumina^®^). After filtration of the OTU table (see Methods section for more details), there were 1 030 109 reads left, assigned to 1 057 OTUs, with a mean of 2 986 reads per sample. The OTUs were assigned to 102 families, 69 orders, 47 classes and 23 phyla. Good’s coverage estimation, used to assess the sampling effort^19^, indicated a mean estimated coverage of 98 ± 2% (minimum of 91% to maximum of 100%). Our main results suggest that: (1) early stage discus larvae gut microbiota is similar to environmental water bacterioplankton, which is mostly composed of *Proteobacteria*; (2) the composition of the microbiota of whole larvae and fry feces is dominated by the genera *Bacteroides* and *Cetobacterium*; (3) the fry feces microbiota taxonomic structure is not significantly different from parental microbiota at 80–100 DPH; (4) the most abundant taxa shaping the structure of the fry gut microbiota originate either from the parents’ skin mucus or environmental water, depending on the number of DPH; and (5) the adult discus cutaneous mucus microbiota structure undergoes a significant taxonomic shift when parents are rearing fry.

### Characterization of the discus gut microbiota ontogenesis

**Figure 1:**
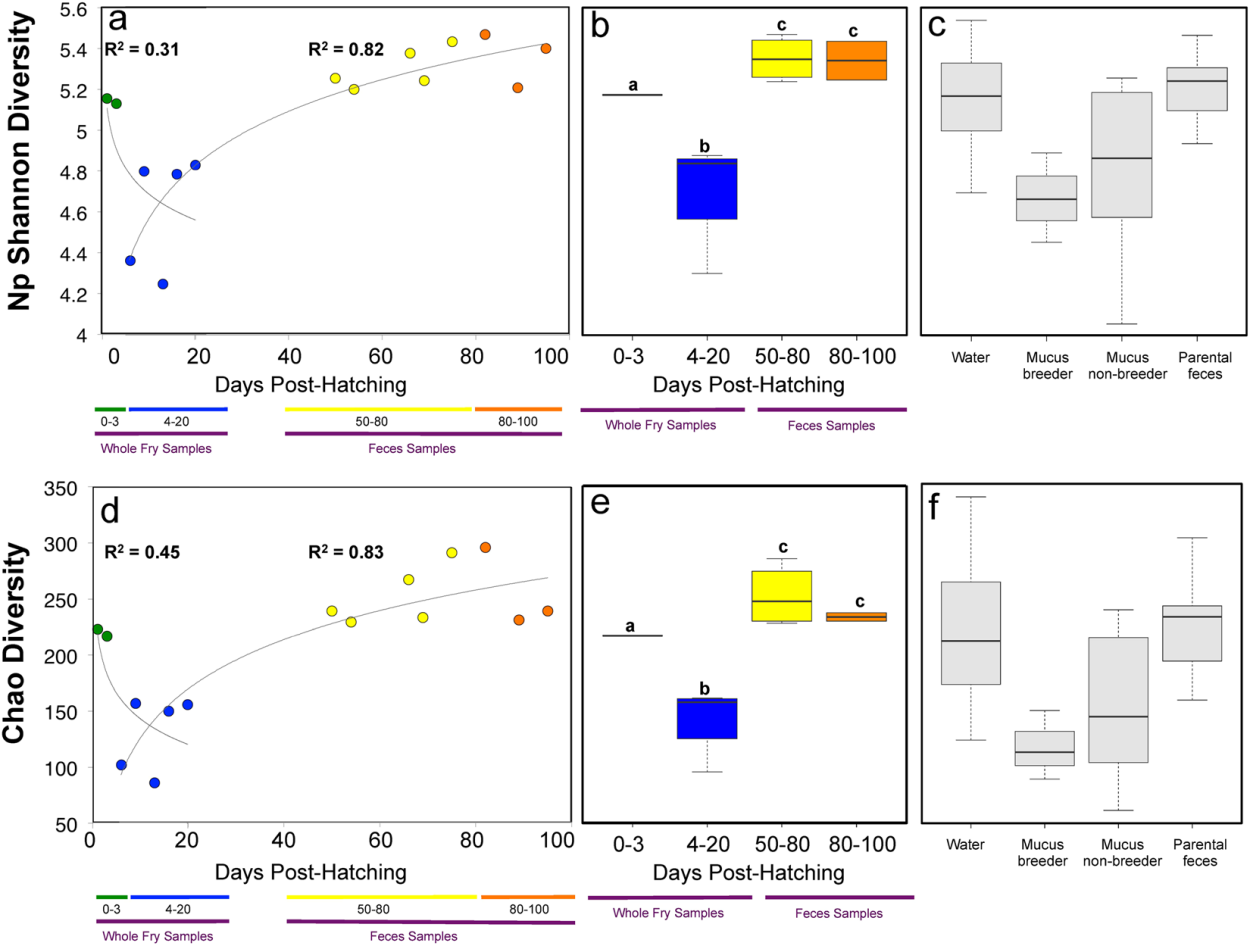
Alpha diversity in whole larvae microbiota significantly decreases between 0–3 and 4–20 DPH, but significantly increases from 4–20 to 50–80 and 80–100 DPH. Non-parametric Shannon (np Shannon) diversity indexes of whole larvae and fry feces microbiota are represented through time in (**a**) (in a scatter plot) and (**b**) (in a boxplot, including significance letters of t-tests at p-value threshold of 0.05). The np Shannon diversity of other microbial niches is represented in (**c**). Chao diversity indexes of whole larvae and fry feces microbiota are represented through time in (**d**) (in a scatter plot) and (**e**) (in a boxplot, including significance letters of t-tests at p-value threshold of 0.05). The Chao diversity of other microbial niches is represented in (**f**). Note that the adult diet is not represented in this figure (neither in **c** nor **f**), as only one sample was collected from this microbial niche. Green markers represent samples taken before mucus feeding (0–3 DPH), blue markers represent samples taken during mucus feeding (4–20 DPH), yellow markers represent samples taken early after mucus feeding (50–80 DPH), and orange markers represent sample taken later after mucus feeding (80–100 DPH). Data layouts in (**a** and **c**) are best explained by logarithmic type trendlines. In (**b** and **c**), y-axis labels are the same than in (**a**). In (**e** and **f**), y-axis labels are the same than in (**d**). Since contrasted patterns of diversity were observed between 0–3 DPH to 4–20 DPH (decrease) and 50–80 DPH to 80–100 DPH (increase), the graphs in **a** and **d** are composites of two separate graphs that were merged together, to better illustrate the contrasted patterns from early to later developmental stages. The composite graphs in (**a** and **d**) were: one diversity scatter plot from 0–3 to 4–20 DPH, and another diversity scatter plot from 4–20 DPH to 50–80 and 80–100 DPH. The logarithmic trendlines were plotted independently on each composite graphs, before merging.

Non-parametric Shannon and Chao1 diversity index showed an overall increasing alpha diversity in fry gut microbiota with DPH (Fig. 1). For graphs in Fig. 1a and d, data layouts were best explained by logarithmic type trend lines with R^2^ correlation value between 0.31 and 0.83. From the developmental stages 0–3 DPH to 4–20 DPH, Shannon and Chao1 diversity significantly decreased (p-value < 0.05). Both diversity index later significantly increased (p-value < 0.05) after mucus feeding (from 4–20 DPH to 50–80 DPH and 80–100 DPH) (Fig. 1a,b,d,e). Minimum values were observed at 13 DPH for Shannon (4.24) and Chao1 (86) diversity while maximum values were at 82 DPH for Shannon (5.46) and Chao1 (296) diversity. In all discus adults’ feces, mean Shannon diversity was 5.4 ± 0.3 and Chao1 diversity was 276 ± 74.

**Figure 2:**
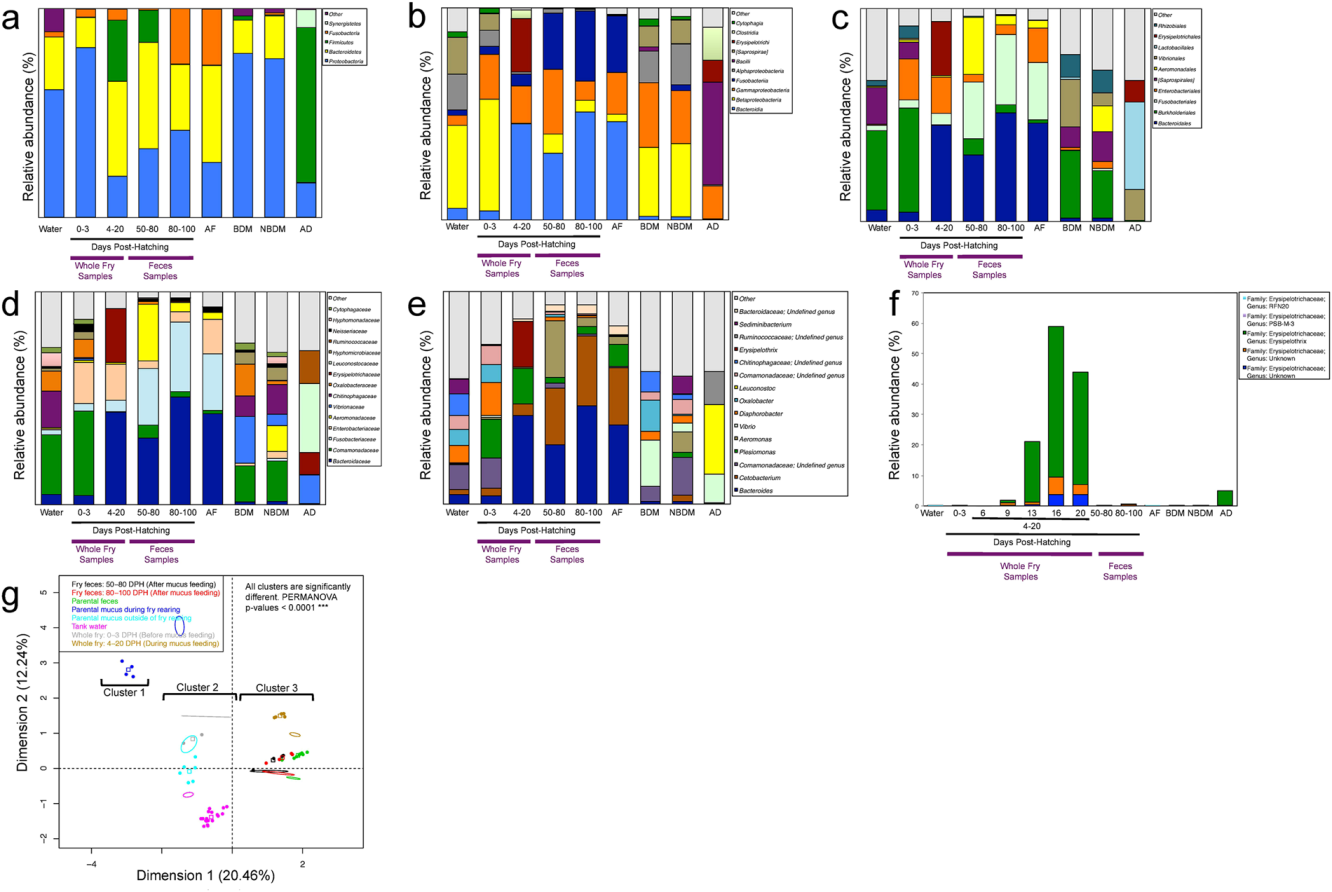
Convergence of whole larvae and fry feces microbial community towards adult-like gut microbiota. The bar charts in (**a**,**b**,**c**,**d** and **e**) respectively represent the relative abundances (%) of the most abundant 5 bacterial phyla, 10 bacterial classes, 10 bacterial orders, 15 bacterial families, and 15 bacterial genera in all samples, in regards to the number of DPH. In this figure, “AF” stands for “Adult feces” (or feces from the discus parents), “BDM” stands for “Breeding discus mucus”, “NBDM” stands for “Non-breeding discus mucus”, and “AD” stands for “Adult diet”. In (**f**), the relative abundances (%) of five OTUs from the *Erysipelotrichaceae* family is shown in regards to the number of DPH and to the different microbial niches studied. Note that in (**f**), the developmental stage from 4–20 DPH is decomposed in its five samplings at 6, 9, 13, 16, and 20 DPH to document more precisely the increased abundance of the *Erysipelotrichaceae* family during this key developmental stage. In (**g**), a Multi-Factorial Analysis (MFA) is shown including all the samples from the different niches (except the sample from Adult diet, since it distorted the representation of all the other samples). The MFA was constructed with the FactomineR package from R, using the relative abundance of the 50 most abundant OTUs in all samples. Confidence ellipses highlight differences at a 0.95 confidence threshold. PERMANOVAs between the three clusters were done on a Thetayc distance matrix, using the vegan package from R, with 10 000 permutations.

The relative abundance of the most abundant bacterial taxa in all sample types (water, adult’s feces, breeding discus mucus, non breeding discus mucus, adult diet, whole larvae, and fry feces) at different DPH, and at all taxonomic levels (Phylum, Class, Order, Family, Genus) are shown in Fig. 2a,b,c,d and e. In Fig. 2e, when the genus was not known, the lowest taxonomic level possible was reported. In water samples, the four most abundant bacterial genera include *Comamonacadeae; Undefined genus* (11.7% of the total bacterial community), *Chitinophagaceae: Undefined genus* (10.1%), *Oxalobacter* (7.6%) and *Comamonadaceae: Undefined genus* (6.5%). In whole larvae before mucus feeding (0–3 DPH): *Plesiomonas* (18.1%), *Comamonadaceae: Undefined genus* (14.1%), *Comamonadaceae: Undefined genus* (8.9%) and *Oxalobacter* (8.5%). Despite the apparent similarity of taxonomic structure between water and whole larvae before mucus feeding (0–3 DPH) (Fig. 2e), PERMANOVAs showed that the microbiota of whole larvae before mucus feeding (0–3 DPH) was significantly different from the surrounding water bacterioplankton community (p-value < 0.0032), regardless of the DPH at sampling time. During the period where fry were feeding on their parents’ skin mucus (4–20 DPH), there was a major increase of the genus *Erysipelothrix* (from 0 to 21.4% of the total fry community), and of the genus *Bacteroides* (from 3.7 to 41.6%), which is the most abundant genus in adult discus gut microbiota. The spike of the genus *Erysipelothrix* is detailed in the bar plot in Fig. 2f, which shows the relative abundance of all genera of the family *Erysipelotrichaceae*, in all sample types. This bar plot also provides a detailed assessment of the relative abundance of these genera through the developmental stage 4–20 DPH (during mucus feeding), and shows that the high abundance of *Erysipelotrichii* between 4–20 DPH is specifically related to a spike of the relative abundance of the genus *Erysipelothrix* (reaching the relative abundance of 49.42% of the total fry microbial community) around 16 DPH. During the same period, there was a major decrease of the class *Betaproteobacteria* (from 39.4 to 0.3% of the total fry gut community), mostly due to the decrease in the family *Comamonadaceae*. In fry feces early after mucus feeding (50 to 80 DPH), the most abundant genera were: *Bacteroides* (27.9%), *Cetobacterium* (26.7%), and *Aeromonas* (26.5%). In fry feces later after mucus feeding (80 to 100 DPH), the most abundant genera were: *Bacteroides* (46.2%), *Cetobacterium* (32.8%), and *Aeromonas* (4.4%). In adult’s feces: *Bacteroides* (37.2%), *Cetobacterium* (26.8%), and *Plesiomonas* (10.7%).

The Thetayc dissimilarity index (TDI) (see Table 1) showed decreasing dissimilarity (therefore an increasing similarity) between fry (whole fry and fry feces) and adult fecal microbiota with increasing DPH. Maximum dissimilarity values were between 0–3 DPH (TDI = 0.8) while minimum values were observed for fry between 80–100 DPH (TDI = 0.42). Contrastingly, TDI between water and whole fry/fry feces showed increasing dissimilarity between the two bacterial niches with DPH: the TDI went from 0.64 (between water and whole fry at 0–3 DPH) to 0.89 (between water and fry feces at 4–20 DPH). PERMANOVAs (Table 2) showed a temporal pattern similar to TDI. Significant differences between fry feces and adult fish fecal microbiota were detected from 50 to 80 DPH (p-values ≤ 0.012), but from 80 to 100 DPH, no significant differences were observed between fry and adult fish fecal microbiota (p-value = 0.08). The same pattern was detected in the Multi-Factorial Analysis (Fig. 2g), made from the relative abundance of the 50 most important OTUs in all samples. Based on the x-axis, which explains the main source of variation (20.46%), we identified 3 clusters of samples that are all significantly different from each other (PERMANONA p-values < 0.0001). The first cluster includes the mucus of breeding discus parents. The second cluster includes: water samples, whole larvae before mucus feeding (0–3 DPH), and mucus of non-breeding discus parents. The second cluster includes: whole larvae during mucus feeding (4–20 DPH), fry feces at 50–80 DPH, fry feces at 80–100 DPH, and adult feces.

**Table 1:** Thetayc Index highlights convergence towards adult-like fecal microbiota and divergence from environmental water bacterioplankton through time. Results of the Thetayc Index analysis comparing the microbial communities of whole larvae and fry feces from different life stages (0–3, 4–20, 50–80, and 80–100 DPH) with the fecal microbiota of their parents. Thetayc indexes were calculated on the mothur platform.

Development Stage	Fry vs. Water			Fry vs. Parent’s Feces		
	Average Thetayc Index	Std Error	Std Deviation	Average Thetayc Index	Std Error	Std Deviation
0–3 DPH, before mucus feeding (whole fry)	0.64	0.08	0.1	0.80	0.08	0.1
4–20 DPH, during mucus feeding (whole fry)	0.89	0.02	0.04	0.71	0.03	0.06
50–80 DPH, after mucus feeding (fry feces)	0.85	0.06	0.1	0.56	0.09	0.2
80–100 DPH, after mucus feeding (fry feces)	0.86	0.04	0.08	0.42	0.1	0.2

**Table 2:** PERMANOVAs highlight maturation of the fry fecal microbiota before 80–100 DPH. Results of the PERMANOVAs (10,000 permutations) comparing the microbial communities of the whole fry or fry fecal microbiota from different life stages with the one in their parents’ feces. Comparisons were only made between similar types of biological samples: whole fry samples compared to whole fry samples, feces samples compared to feces samples, and skin mucus samples compared to skin mucus samples. PERMANOVAs were performed with package vegan on R. “df” stands for “degrees of freedom”.

	Comparison		df (residual)	F-value	p-value
Fry	Whole fry microbiota from 0–3 DPH (before mucus feeding)	Whole fry microbiota from 4–20 DPH (during mucus feeding)	5	3.2363	0.04762*
	Fry fecal microbiota from 50–80 DPH (after mucus feeding)	Fry fecal microbiota from 80–100 DPH (after mucus feeding)	5	0.9723	0.4857 NS
	Fry fecal microbiota from 50–80 DPH (after mucus feeding)	Parents’ feces	9	3.4198	0.0121*
	Fry fecal microbiota from 80–100 DPH (after mucus feeding)	Parents’ feces	9	2.0718	0.07469 NS
Parents	Mucus microbiota when feeding fry	Mucus microbiota when not feeding fry	10	1.5933	0.0384*

### Interactions between the main microbial reservoirs and the fry gut microbiota

**Figure 3:**
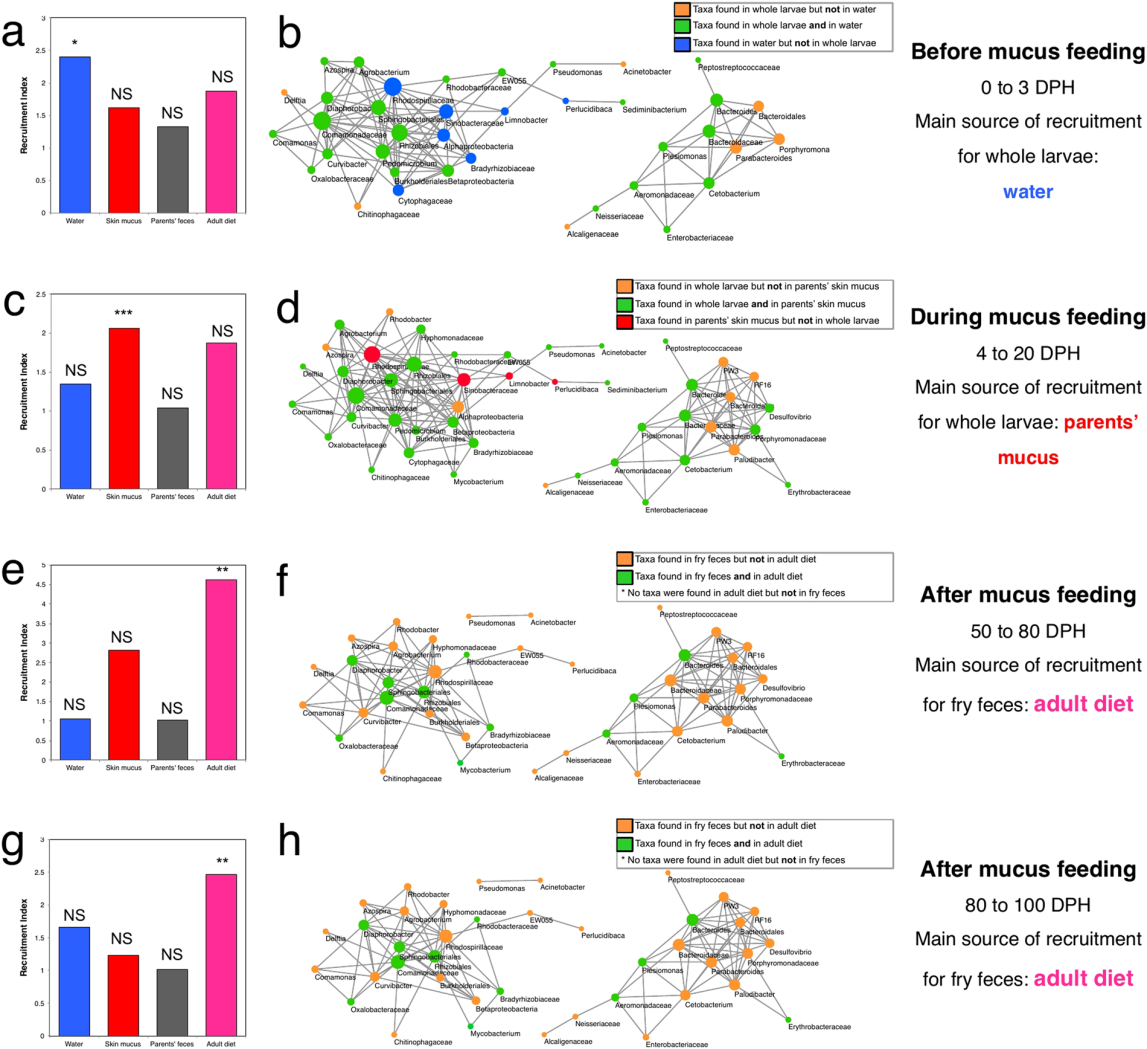
Microbial taxa from water and skin mucus are drivers of whole fry and fry feces microbiota. In (**b**,**d**,**f** and **h**) co-abundance based networks of the 50 most abundant OTUs, constructed with Cytoscape v. 3.2.1. Each dot (node) and its label represent an OTU defined at the lowest level possible. A link (edge) between two nodes highlights a Spearman correlation index >0.9 between the two taxa and a p-value < 0.0001 (corrected with Bonferroni). The size of each node is proportional to the number of edges that it is connected to. In (**a**,**c**,**e** and **g**) bar charts representing the recruitment index (see Methods section for details on calculation) from each of the environmental microbial reservoirs (water, parents’ mucus, adult diet), at the four life stages of the fry: before mucus feeding (0–3 DPH), during mucus feeding (4–20 DPH), early post-mucus feeding (50–80 DPH), and late post-mucus feeding (80–100 DPH). Recruitment from water is represented by blue bars, recruitment from skin mucus is red, recruitment from parental feces is grey, and recruitment from the adult diet is pink.

The microbial recruitment indexes for each of the main four potential microbial reservoirs (environmental water, parent’s skin mucus, parent’s feces, or adult diet), by the fry at each of their main developmental stage (0–3, 4–20, 50–80, and 80–100 DPH) are shown in Fig. 3 (a,c,e,g). Before mucus feeding (0–3 DPH), microbial taxa from the water were significantly recruited (p-value = 0.042). During mucus feeding (4–20 DPH), microbial taxa from the parental mucus were strongly significantly recruited (p-value < 0.00001). Early after mucus feeding (50 to 80 DPH) and later after mucus feeding (80–100 DPH), only microbial taxa from the adult’s diet were significantly recruited (p-value < 0.01).

Microbial taxa co-abundance networks were constructed for the four developmental stages investigated (0–3 DPH, 4–20 DPH, 50–80 DPH, and 80–100 DPH), based on the relative abundance of the 50 most abundant OTUs in all the dataset. Each node of the network represents a bacterial OTU, identified at the lowest possible taxonomic level. At each developmental stage, the nodes of the network were colored according to their provenance (e.g. in Fig. 3a, OTUs that are found in water samples but that are not found in whole fry samples were colored in blue). For representation purposes, only the OTUs found in the microbial reservoir from which there was a significant recruitment of microbial taxa by the fry (Fig. 3 b,d,f,h) were considered. A connection between any OTU pair, resulting from a co-variation of relative abundance, is interpreted as a taxonomic interaction^20^. First, these networks highlight complex interactions between bacterial taxa from the main microbial reservoirs and the fry gut (whole larvae and fry feces) microbiota. Many of these interactions are indirect: for instance, in Fig. 1b an OTU of the *Cytophagaceae* family found in water is connected with an OTU of the *Comamonadaceae* family (which is one of the most important OTU structuring the network, because of its numerous connections to other OTUs) shared between whole larvae and water, which in turn co-varies with an OTU of the *Chitinophagaceae* family only found in whole larvae microbiota. Thus, bacterioplankton-associated OTUs have significant indirect interactions with whole-larvae-associated OTUs. Second, these networks highlight a specialization of the fry gut microbiota (either whole larvae or fry feces) through increasing DPH. Indeed, not only the number of taxa unique to the fry microbiota increases with DPH, but also, the number of connections between these fry symbionts increases from pre-mucus feeding (0–3 DPH) to mucus feeding stage (4–20): before mucus feeding (0–3 DPH), 7 taxa were unique to the whole larvae, with an average of 2.86 inter-taxa connections; 9 taxa during mucus feeding (4–20 DPH), with an average of 7.25 inter-taxa connections; 28 taxa early after mucus feeding (50–80 DPH), with an average of 5.8 inter-taxa connections; and 28 taxa later after mucus feeding (80–100 DPH), with an average of 5.8 inter-taxa connections.

### Parental-behavior-associated skin microbiota reshaping on adult fish

**Figure 4:**
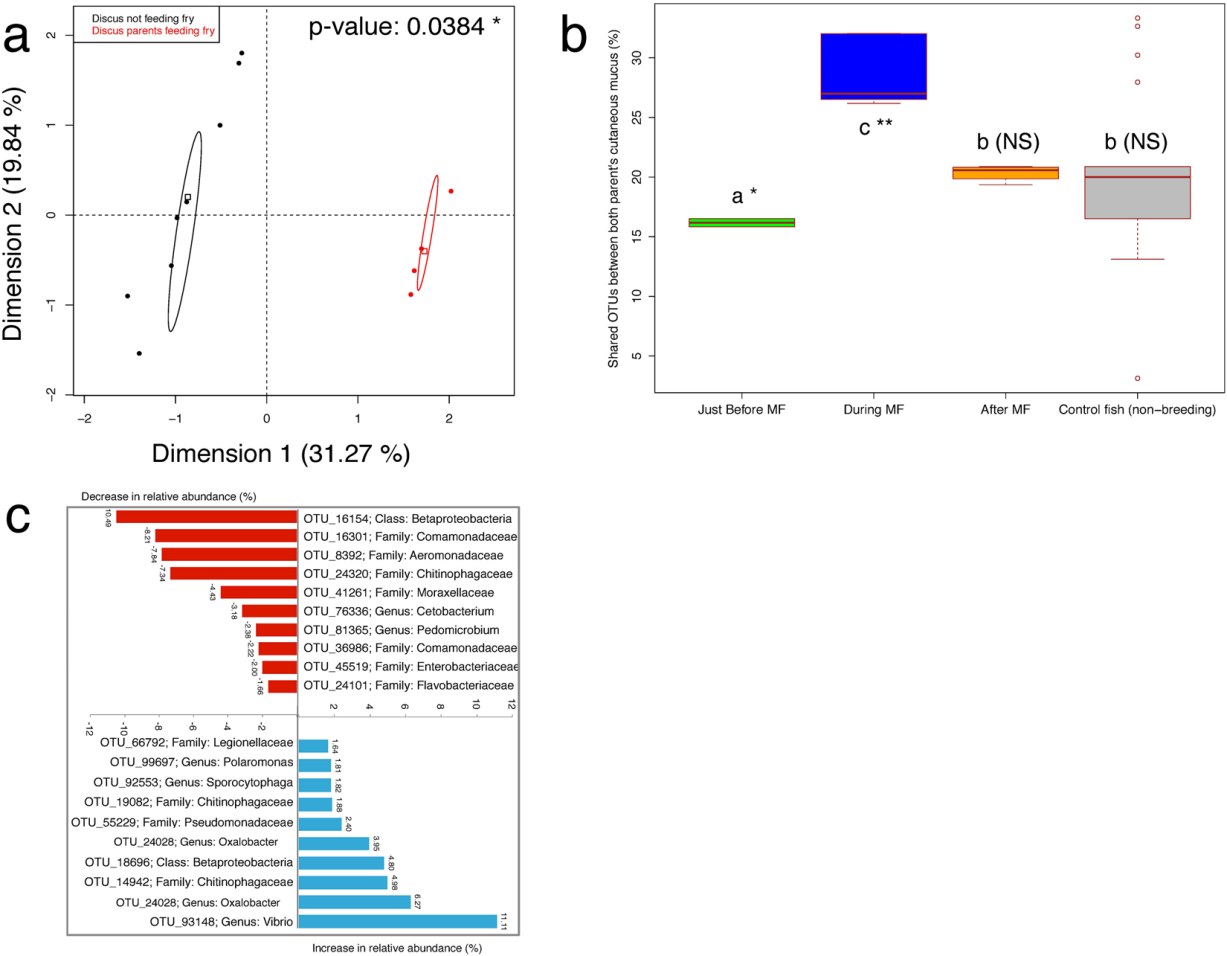
Taxonomic shift towards a core skin mucus microbiota on parents rearing fry. In (**a**) Multi-Factorial Analysis (MFA) based on the variations of relative abundance of bacterial taxa (identified at the lowest taxonomic level possible) in regards of the reproductive status of the discus: breeding or not-breeding. Confidence ellipses highlight significant differences at a 0.95 confidence threshold. In (**b**) boxplots representing the percentage of shared OTUs between both parents of the breeding couple before mucus feeding (MF), during mucus feeding, after mucus feeding, and between 20 random couples of non-breeding control fish. In (**c**) analysis of the taxonomic shifts that occurred in the skin mucus microbiota of parents when getting into reproduction phase. Red bars represent a decrease while blue bars represent an increase (in % of relative abundance). Each OTU was labeled at the lowest taxonomic level possible.

The multifactorial analysis (MFA) shown on Fig. 4a revealed significant differences (non-overlapping confidence ellipses) in terms of taxonomic structure between the skin mucus microbiota of non-breeding fish versus parenting fish (breeding fish rearing fry). PERMANOVAs confirmed the significant difference between these groups (p-value = 0.0384). No significant difference was observed between cutaneous mucus microbiota of breeder fish when not rearing fry (at T0) and control fish (p-value = 0.3). The MFA indicated that a total of 51.11% of the data layout is explained by the two groups (non-breeding fish and parenting fish). In Fig. 4c, we documented which were the OTUs that were mostly affected by the taxonomic restructuring taking place when discus parents started rearing fry. The OTUs for which relative abundances were the most negatively affected by the mucus feeding behavior were OTUs (identified at the lowest taxonomic level possible) from the class *Betaproteobacteria* (−10.40%), in the family *Comamonadaceae* (−8.21%), and *Aeromonadaceae* (−7.84%). Contrastingly, OTUs for which relative abundances were the most positively affected by the mucus feeding behavior were OTUs in the genus *Vibrio* (+11.11%), in the family *Oxalobacter* (+6.27), and *Chitinophagaceae* (+4.98%). Furthermore, Fig. 4b shows that the relative abundance of shared OTUs between the skin mucus of both parents from the breeding pair increased significantly (p-value < 0.01) from 16.2 ± 0.3% to 29 ± 3% when discus parents started feeding their brood. After the end of parental skin mucus feeding, the relative abundance of shared OTUs between the skin mucus of both parents from the breeding pair decreased significantly (p-value < 0.01) from 29 ± 3% to 20.3 ± 0.6%, which is not significantly different than the relative abundance of shared OTUs between 20 random pairs of control (non-breeding) adult discus (average of 20 ± 7%).

## Discussion

The establishment of a mature (adult-like) stable microbiota is a process influenced by numerous interactions between the bacterial consortia, the developing host and the stochasticity of environmental conditions. Overall, gut, cutaneous mucus, and bacterioplankton’s ecological niches are not isolated environments: they constitute a network of interrelated communities experiencing perpetual exchange^21^ driven by both neutral (drift) and deterministic factors (host genotype selection, microbe-microbe interactions, active dispersal)^9^. In most fish, neutral factors are mainly driving the microbiota assembly during larvae and juvenile developmental stages, whereas in mammals, the vertical transmission of a maternal core microbiota via breastfeeding and (to a less extent) vaginal delivery “accelerates” the stabilization and maturation of the infant gut microbiota^22^. While investigating the discus fry gut microbiota ontogeny, we observed very contrasted patterns of colonization: in early developmental stages, whole larvae before mucus feeding (0–3 DPH) harbored bacterial consortia similar in composition and diversity to environmental water bacterioplankton communities (Figs 1a–f and 2a–e,g), as observed in early stages of other fish species^10^. For instance, in these niches *Proteobacteria* was by far the dominant phylum, with a consistent relative abundance between 60 to 83% in all discus fry and environmental samples. Contrastingly, at later developmental stages (4–20, 50–80, and 80–100 DPH) the relative abundance of *Proteobacteria* in whole fry and fry feces communities drastically dropped from 84% to 19% while it remained high (≈60%) in environmental samples. Our observations are consistent with a recent study^10^, which documented a high abundance of *Proteobacteria* in the surrounding environment and in the gut microbiota of early stage zebrafish larvae. It is possible that the prevalence of *Proteobacteria* at the onset of gut microbiota ontogenesis is related to the opportunistic nature of several strains in this phylum^18, 23^ that can colonize the gut in early development, when weak colonization resistance is offered by commensal gut symbionts^24^. This resistance increases later during development^23^, as the gut is colonized by a more specific microbiota.

Then, as soon as fry started mucus-feeding on their parents (4–20 DPH), major taxonomic shifts of the gut microbiota composition of the fry occurred (Fig. 2). Indeed, the *Alphaproteobacteria*, *Betaproteobacteria* and *Gammaproteobacteria* that composed most of the fry gut community before mucus-feeding were eclipsed by symbionts from the classes *Bacteroidia* and *Erysipelotrichi* just as mucus-feeding behavior began. Such a colonization pattern mimics what has been documented in mammals. Indeed, in humans, environmental, vaginal and skin bacteria usually do not settle permanently in the newborn’s gut^25^: they are quickly replaced by bacteria from breast milk. The abundant class *Bacteroidia* is part of the *Bacteroidetes* phylum, which have been documented in the gut microbiota of several mammals (reviewed in ref. 26), plays roles in the normal development of the gastro-intestinal tract (GIT)^27^, the activation of T-cell mediated responses^28^ and resistance to pathogen colonization^29^. Interestingly, the relative abundance of the class *Erysipelotrichi* in the fry gut increased drastically (from 0 to 24.9%) during skin mucus feeding, and then dropped to 1% after weaning. While its abundance increased in fry gut (Fig. 2), it did not increase in parental skin mucus (Fig. 4c) during this period. Furthermore, this increase in *Erysipelotrichi* was mostly related to a spike of the genus *Erysipelothrix* at 16 DPH. Consequently, our results show that an OTU from this genus is one of the most highly recruited OTU from the parental skin mucus (Table 3), although its abundance in parental mucus was very low (Fig. 2f). This poorly known taxa has been reported in the gut microbiome of fish^30^, rhinoceros beetle (*Trypoxylus dichotomus*)^31^ and humans with a high-fat diet^32^. A review from Kaakoush (2015)^33^ suggests that members of *Erysipelotrichaceae* might have important roles to play in lipid metabolism of the host: *Erysipelotrichaceae* is peripherally related to the superfamily *Lachnospiraceae* ^34^, which are producers of butyrate, a major energy source for the colonic epithelium^35^. However, bacteria from this taxa are also known to significantly correlate to several metabolic diseases (review in ref. 33). Therefore, we cannot confirm whether the relative abundance increase of members of the *Erysipelotrichaceae* (especially *Erysipelothrix*) in our study is related to a potential role played by this taxa in the lipid metabolism in the fry gut, or if this increase is merely due to the weak colonization resistance offered by commensal gut symbionts at early developmental stages^24^.

**Table 3:** Potential vertical transmission of microbial symbionts from parents’ skin mucus to fry through skin mucus feeding behavior (4–20 DPH). Taxa in this table: (1) are shared between breeding discus skin mucus and whole larvae microbiota during mucus feeding (4–20 DPH); (2) are absent from environmental water bacterioplankton; and (3) have a higher relative abundance in whole larvae during mucus feeding (4–20 DPH) than in breeding discus skin mucus, highlighting the differential niche preference of these microbial taxa. In this table, “WL” stands for “Whole Larvae”, “NBDM” stands for “Non-breeding discus mucus”, and “BDM” stands for “Breeding discus mucus”.

OTU_ID	Taxonomy (lowest taxonomic level possible)	Mean relative abundance in NBDM (%)	Mean relative abundance in BDM (%)	Mean relative abundance in WL (4–20 DPH) (%)	Ratio of relative abundance in WL/BDM
OTU_68244	***Erysipelothrix***	**0.0506**	**0.0056**	**19.4440**	**3446.74**
OTU_20327	*Erysipelothrix*	0	0.0012	0.5776	485.37
OTU_33665	*Erysipelothrix*	0	0.0036	0.7430	209.09
OTU_45519	***Plesiomonas shigelloides***	**2.6736**	**0.0047**	**15.9445**	**3416.26**
OTU_81538	*Bacteroides*	0.0080	0.0012	0.4107	345.14
OTU_68201	*Bacteroides*	0.0700	0.0012	0.2159	181.42
OTU_4007	*Bacteroides uniformis*	0.0084	0.0027	0.5708	210.42
OTU_67054	*Bacteroides uniformis*	0.0028	0.0023	0.3523	150.96
OTU_27399	*Peptostreptococcaceae*	0.0196	0.0014	0.7987	588.40
OTU_16157	*Rhodobacteraceae*	0.1130	0.0053	0.1144	21.41

After weaning (from 21 DPH), discus fry started eating the adult’s diet. This dietary change correlated with an important increase of the relative abundance of the genus *Cetobacterium*, which is the second most abundant taxa of the adult microbiota (Fig. 2e). From then, the high relative abundance of adult-associated genera *Cetobacterium* and *Bacteroides* persisted into the mature gut microbiota (50–80 DPH to 80–100 DPH). In human infants, the same phenomenon was observed: weaning shifts the gut microbiota towards an adult-like composition due to the loss of maternally provided immunological factors and the removal of breast-feeding-derived bacterial symbionts^36^. Additionally, we observed that weaning from cutaneous mucus-feeding translated into a significant diversification of the gut microbiota, just like weaning from breast-milk in human neonates. Likewise, as soon as the discus fry stopped mucus feeding and started eating solid foods, the Shannon and Chao1 diversity index of their gut microbial community significantly increased. Such colonization pattern is consistent with what is generally observed in humans^37^, but contrasts with what was reported in zebrafish^10^ and in three other fish species^38^, in which gut microbiota diversity decrease significantly over the course of development^10^. It remains possible that differences observed between pre and post-weaning discus might be mitigated by the two different sampling strategies (whole larvae *versus* fish feces), as a whole juvenile fish microbiota is expected to be more diversified than feces only (more microbial niches are present on a whole juvenile fish than only in the fish gut). Thus, the pattern of increasing alpha diversity with DPH–as observed in our study–is conservative. Interestingly, a similar pattern of increasing alpha diversity during gut microbiota ontogenesis was reported in damselfish^39^, thus suggesting that fish gut microbiota patterns of alpha diversity during ontogenesis are species-specific.

Our results further contrast with reports of the zebrafish gut microbiota ontogenesis concerning the maturation time of the microbiota (the time needed for the fry microbiota to become similar to their parent’s). At the developmental stage 80–100 DPH, the fry gut community is not significantly different from the adult gut microbiota, suggesting that microbiota composition reached maturity. However, as early as 50–80 DPH we documented prevalence and steadiness of adult microbiota characteristic genera (*Cetobacterium* and *Bacteroides*). Fifty DPH is proportionately early in development when compared to the fast-developing zebrafish, in which the adult-like microbiota is not prevalent before sexual maturity, which occurs at 75 DPH^10^. Contrastingly, the discus fish only reaches sexual maturity at about two years after hatching (730 DPH). Taken together, these results suggest that skin-mucus-feeding could potentially stimulate the quick maturation of the discus gut microbiota, by helping funnel the larval microbiota to adult-like communities. In humans, a similar pattern is observed: the adult-like gut microbiota is reached around 500 days after birth, whereas sexual maturity is only reached at 11–12 years of age^40^. Consequently, many of our results (the post-weaning diversification, the increasing diversity with DPH, and the fast maturation of the gut microbiota) suggest that discus fish have a mammalian-like gut microbiota ontogeny.

This conclusion is also supported by the results of the recruitment analysis (Fig. 4a,c,e,g), which suggest that at early developmental stages (before mucus feeding, 0–3 DPH), the majority of microbial taxa are recruited from environmental water (Fig. 4a), but a few days later (during mucus feeding, 4–20 DPH), microbial taxa are mostly recruited from parental skin mucus. The network analysis (Fig. 4d) suggests that several microbial taxa from parental mucus interact and are shared with whole larvae microbiota. For instance, the most abundant OTUs in whole larvae during mucus feeding (4–20 DPH) are from the genera *Cetobacterium*, *Plesiomonas*, and *Bacteroides*, which are all shared with parental skin mucus (Figs 2e and 4d). Our results from Table 4 suggest that the taxa which are documented in whole larvae during mucus feeding (Table 4), and that are potentially recruited from parental skin mucus include the members of the following clades (identified at the lowest taxonomic level possible): *Erysipelothrix*, *Plesiomonas shigelloides*, *Bacteroides*, *Bacteroides uniformis*, *Peptostreptococcaceae*, and *Rhodobacteraceae*. Figure 4d shows that some of these taxa interact directly or indirectly with other abundant OTUs of the whole larvae microbiota during mucus feeding (4–20 DPH). Furthermore, our results suggest that taxa settling permanently in fry guts (at least until 80–100 DPH) (Table 4), and potentially originating from parental skin mucus, include members of the following clades (identified at the lowest taxonomic level possible): *Diaphorobacter*, *Comamonadaceae*, *Enterobacteriaceae*, *Oxalobacter*, *Vogesella*, and *Comamonas*.

**Table 4:** The most abundant fecal microbial symbionts of the fry after mucus feeding (80–100 DPH) are differentially shared between the fry and three microbial niches which are potential sources of symbionts for inoculation of the fry microbiota during development: breeding discus mucus, adult diet, and environmental water. In this table, the fry development stage at 80–100 DPH was used instead of fry at 50–80 DPH, because its taxonomic structure is non-significantly different from the adult fecal microbiota. The relative abundance of each taxa was printed in bold in the potential microbial source (breeding discus mucus, adult diet, and environmental water) where they were the most abundant. The parental feces were included in this table as a comparison to fry feces at 80–100 DPH. Parental feces were not considered as a potential microbial source, as the recruitment index from this niche was not significant at any of the developmental stages (0–3, 4–20, 50–80, 80–100 DPH), see Fig. 3. Non-breeding discus mucus is intentionally not included in this table as it is not a potential source of microbial symbionts for the fry. In this table, “BDM” stands for “Breeding discus mucus”.

OTU taxonomy (lowest level possible)	Relative abundance in fry feces after mucus feeding (80–100 DPH) (%)	Relative abundance in parental fecal microbiota (%)	Relative abundance in BDM (4–20 DPH) (%)	Adult diet	Relative abundance in water (0–3 DPH) (%)
*Bacteroides*	46.24	37.21	1.26	0.19	**5.80**
*Cetobacterium*	32.84	26.80	0.05	0.00	**2.30**
*Aeromonadaceae*	4.38	3.40	0.03	0.03	**1.00**
*Bacteroidaceae*	4.01	4.13	0.01	0.00	**0.50**
*Plesiomonas*	3.54	10.73	0.02	0.08	**0.21**
*Neisseriaceae*	1.16	1.85	0.05	0.00	**0.19**
*Alcaligenaceae*	1.13	0.20	0.00	0.00	**0.15**
*Diaphorobacter*	0.85	0.33	**3.96**	0.06	2.23
*Comamonadaceae*	0.83	0.51	**6.93**	0.13	3.44
*Enterobacteriaceae*	0.70	4.69	**1.20**	0.00	0.38
*Comamonadaceae*	0.62	0.33	**3.60**	0.00	1.19
*Erysipelotrichaceae*	0.38	0.09	0.00	**2.28**	0.10
*Rhodobacter*	0.34	0.00	0.00	0.00	**0.61**
*Enterobacteriaceae*	0.34	0.57	0.00	0.00	**0.02**
*Oxalobacter*	0.24	0.07	**14.96**	0.09	0.21
*Bacteroidaceae*	0.24	1.40	0.00	0.00	**0.07**
*PW3 (Rikenellaceae)*	0.18	0.36	0.00	0.00	**0.41**
*Peptostreptococcaceae*	0.16	0.10	0.01	0.00	**0.05**
*Vogesella*	0.16	0.03	**0.74**	0.00	0.55
*Comamonas*	0.13	0.06	**0.27**	0.00	0.07

In the literature, species-specific bacterial symbionts have been shown to provide a vast range of beneficial molecules and services to their host: biosynthesis of vitamins, amino acids, enzymes, antimicrobial compounds and isoprenoids^41^, immune system maturation^42^, nutrients absorption^43^, gut motility and differentiation^44^, lipid metabolism^45^, as well as synergistically interacting with the host immune system to control opportunistic pathogens^25^. By foraging on their parent’s skin mucus, it is likely that fry neutrally recruit specific bacterial taxa that are beneficial for their growth. For instance, our results show that OTUs from the genera *Oxalobacter* and *Bacteroides* are shared between breeding discus mucus and whole larvae microbiota (4–20 DPH). Both of these genera have been reported to play important roles for the gut metabolism in humans: strains of *Oxalobacter* can degrade oxalate^46^, while strains of *Bacteroides* can improve metabolic and immunological dysfunction^47^.

The hypothesis that discus fish are recruiting specific microbial strains from their parent’s skin mucus is further supported by the taxonomic shift of the parent’s skin mucus microbiota when rearing fry. Other studies have documented changes in proteins, hormones, immunoglobulins and lectins profiles of skin mucus when parents went into reproductive phase^12, 14–16^. Consequently, these changes also impact the resources and habitat available for the growth of microbial symbionts of the skin mucus. Since other studies have shown that the composition of the endogenous microbiota is affected by the types and quantity of hormones secreted by the host^48^, we expected to observe a taxonomic shift when the discus parents went into reproductive phase. However, given the extent of our current results, we cannot determine exactly whether the observed taxonomic shift can be attributed to a change of environmental resources and habitat for skin mucus symbionts, or to a mechanism selected through the evolution of the discus fish to promote a vertical transfer of specific pioneering microbial strains to the fry. Nevertheless, this last hypothesis is partly supported by the significant increase in the ratio of shared/unique OTUs in cutaneous mucus microbiota of parenting discus compared to non-breeding adults (Fig. 4b). These results suggest that there is a core microbiota associated with the cutaneous mucus of parenting discus fish, as it is observed in mammalian milk. To this respect, Hunt *et al*.^11^ observed a core microbiota of 9 OTUs associated with milk of breast-feeding human mothers. In mammals, vertical transmission of a species-specific core microbiota is crucial to the progeny, as it provides endogenous symbiotic bacteria that synthetize beneficial molecules (fatty acids, vitamins, amino acids, enzymes, antimicrobial compounds) and exert numerous functions and services such as immune system maturation and stimulation and protection against opportunistic pathogens (reviewed in ref. 5).

Overall, our results suggest that (1) before *contacting* (0–3 DPH), discus fry microbiota show colonization patterns dominated by microbial taxa acquired horizontally, a characteristic of the GIT microbiota ontogeny of other fish such as the zebrafish; (2) when *contacting* (4–20 DPH), the vertical recruitment of microbial symbionts from skin mucus correlates with a switch to a mammalian-like gut microbiota ontogenesis; (3) discus experience a taxonomic shift in their cutaneous mucus microbiota composition when rearing fry, potentially in order to vertically transfer a core microbiota to their offspring. In the future, studies targeting discus as an alternate fish model for gut microbiota ontogenesis should use shotgun metagenome, or metatranscriptome approaches to characterize accurately the functional properties of the pioneering strains transmitted vertically and horizontally.

## Methods

### Fish rearing

All fish were reared at the Laboratoire régional des sciences aquatiques (LARSA) at Université Laval (Quebec, QC). 15 adult discus (average lenght ≈12.5 cm) fish were obtained from Discus Paradise (http://www.discusparadise.com/) in Montreal (QC), an official distributor of aquacultured Stendker Discus (http://diskuszucht-stendker.de/) from Germany. All fish were acclimated during eight weeks in a bare 1040 L tank before the start of the experiment with the following water conditions: pH = 6.5, 14/10 h photoperiod, [dissolved O_2_] = 7.0–7.9 mg/L (>90% saturation), water temperature = 32 °C and conductivity = 130–150 µs/cm. Six 120 L tanks were interconnected to the acclimatization tank. Of these, three were used to house potential adult breeding pairs and three were used to raise the discus juveniles. All tanks were connected to a central filtration system consisting of a 200 L biofilter and a 96.3 L STA-RITE sand filter (model S8S70). All tanks were siphoned daily to remove feces and leftover food from the tank’s bottom. A daily water change of 25% volume of the system water was done with an input mix of 2/3 treated tap water and 1/3 deionized water. Fish were fed until satiation two times daily with a homemade mixture as described in Giovanetti and Lucanus (2005)^49^. All fish were identified using natural morphological markings.

### Ethics statement

This project and protocol were approved by the “*Comité de protection des animaux de l’Université Laval* “ (CPAUL) (protocol number 2013159-1). All methods were carried out *in accordance with* the approved guidelines.

### Discus breeding

After the eight weeks acclimatization period, all adult fish were left in the same 1040 L tank to identify potential breeding pairs. Four breeding cones made of PVC pipes were added to the tank. To trigger breeding behavior, the water temperature was lowered from 32 °C to 28 °C for 48 h and was then increased back to 32 °C to simulate temperature shifts induced by a heavy rainfall. Breeding behavior was defined as (1) two fish protecting a delimited area around a cone; (2) courtship between two fish of different sex; or (3) a pair spawning on a breeding cone. Three potential breeding pairs were isolated in breeding compartments and one of these pairs successfully raised offspring. All other adult fish (N = 9) were used as controls. We acknowledge that the number of breeding pairs (N = 3) and progeny documented (N = 1) is limited, due to the high market price of discus fish, and the numerous challenges associated with keeping and breeding this very delicate species. To palliate the limited number of biological observations, three technical replicates were prepared and sequenced for every biological sample (PCRs and sequencing were done separatedly for each replicate, but replicates of the same sample were pooled for downstream analysis). Once the potential breeding pair were identified, they were moved to a 120 L breeding tank containing a breeding cone. After spawning, the pair ventilated their eggs for two days. Then, the non-swimming fry hatched and stayed attached to the cone for 48 hours. During this time, parents still ventilated their fry and the larvae fed on their yolk sacs. After 48 hours, 85 healthy discus larvae started swimming and instinctively feeding on both of their parent’s skin mucus for three weeks. The fry were kept with their parents until 20 days post-hatching (DPH) and were then moved to a 120 L rearing tank. In rearing tanks, fry were fed with the same feed as the adult fish.

### Sampling on adult fish

Skin mucus was sampled on adult fish (including the breeding pair and the controls) (1) at T0 (after the acclimatization period); (2) at two DPH of the fry; (3) at 14 DPH; and (4) at 50 DPH. Skin mucus sampling was performed by gently rubbing a sterile cell scraper (Sarstedt) on ≈ 50% of the surface of the right side of each fish. Skin mucus samples were stored at −80 °C in 1.5 mL Eppendorf tubes.

Feces were sampled on adult fish (including breeders and controls) (1) at T0 (after the acclimatization period); (2) at each day fry or fry feces were sampled: day 0 post-hatching (eggs) and days 2, 6, 9, 13, 16, 20, 50, 54, 66, 69, 75, 82, 89, 96, and 100. Feces samplings were performed while observing the adult fish, by siphoning fecal matter released by the adult fish shortly after eating, before it came in contact with other feces (e.g. fry feces) at the bottom of the tanks. To isolate fecal microbiota from residual water bacterioplankton, feces were rinsed five times with 5 mL of sterile PBS 1 M and three times with 5 mL of sterile MilliQ water before being stored at −80 °C in 1.5 mL Eppendorf tubes.

### Sampling on fry

Eggs (N = 10) were sampled one day after spawning. 10 whole discus larvae were sampled at 2, 6, 9, 13, 16, 20 days (every four days) after hatching. Whole larvae had to be sampled to characterize intestinal microbiota, since the very small size of the fry (3–10 mm length) prevented us from dissecting individual larvae and the presence of the parents in the same tank as the fry prevented us to siphon specifically the fry feces from the tank’s bottom. Furthermore, fry before (0–3 DPH) and during (4–20 DPH) mucus feeding were too small to dissect without potentially introducing important bias or contamination in the samples. To isolate gut microbiota from residual water bacterioplankton and cutaneous mucus microbiota, larvae and eggs were thoroughly rinsed five times with 5 mL of sterile PBS 1 M and three times with 5 mL of sterile DNA-free MilliQ water before being stored at −80 °C in 1.5 mL Eppendorf tubes. This method is adapted from Bakke *et al*.^50^. At days 50, 54, 66, 69, 75, 82, 89, 96, and 100 after hatching, feces were sampled by siphoning the bottom of the fry tank shortly after eating. All fry feces’ samples were pooled at each sampling. From 50 DPH, fry feces were sampled rather than whole fish since (1) the bigger size of the fry (average length ≈2 cm) enabled us to collect substantial fecal matter; (2) the fry were separated from their parents; and (3) we had to keep the 15 juveniles that were left from previous samplings for the longer-term samplings (until 100 DPH). Fry and juvenile discus feces were rinsed and stored with the same method as previously described for adult feces.

### Water sampling

Water samples were collected at each adult or fry sampling: T0 and 0, 2, 6, 9, 13, 16, 20, 50, 54, 66, 69, 75, 82, 89, 96, and 100 DPH. Three liters of system water were sampled in sterile Nalgene™ bottles at each sampling. Water samples were filtered on 0.2 μm membranes (Nucleopore^**©**^) using a Masterflex Easy-Load^®^ II peristaltic pump from Cole-Parmer^®^. Post-filtration, the membranes were stored dry at −80 °C.

### Sample processing

DNA extraction of skin mucus samples, whole larvae, and 0.2 μm membranes from water samples was performed using DNeasy^®^ Blood and Tissue Kit from QIAGEN according to the manufacturer’s instructions. DNA extraction of fecal samples and adult diet was performed using QIamp^®^ Fast DNA Stool Mini Kit according to the manufacturer’s instructions. Extracted DNA from feces, skin mucus, whole larvae, adult diet, and water was stored at −80 °C until amplification. DNA was amplified using a nested polymerase chain reaction (PCR) approach adapted from Boutin *et al*.^51^. In the first PCR, the complete 16S rRNA gene was amplified using the forward primer F-Tot of sequence 5′-GCAGGCCTAACACATGCAAGTC-3′ and the reverse primer 1389R of sequence 5′-ACGGGCGGTGTGTACAAG-3′. In the second PCR, the region V4 of the 16S rRNA gene was targeted with forward primer S-D-Arch-0519-a-S-15 of sequence 5′-CAGCMGCCGCGGTAA-3′ and the reverse primer S-D-Bact-0785-b-A-18 of sequence 5′-TACNVGGGTATCTAATCC-3′. 1 μL of PCR product from the first PCR diluted [1/10] with 9 μL of QIAGEN’s Microbial DNA Free Water was used as template for the second PCR. All PCR reactions were performed according to the manufacturer’s instructions of Q5^®^ High-Fidelity DNA Polymerase from New England BioLabs^®^ Inc. To increase precision in the assessment of microbial community composition and to reduce PCR bias, PCRs were done in triplicates. All three PCR products for each sample were kept separate post-PCR and were labeled as technical replicates, which were individually sequenced (note that for downstream bioinformatic analysis, technical replicates were later pooled after sequencing for statistical accuracy). PCR program: (1) 30 sec 98 °C; (2) 10 sec 98 °C; (3) 30 sec 64 °C; (4) 20 sec 72 °C; (5) 2 min at 72 °C; 35 amplification cycles total. Amplified DNA was purified by extracting the V4 DNA fragment (≈ 250 base pairs) on a [1.5%] agarose gel. Gel extraction was performed using QIAquick Gel Extraction Kit from QIAGEN according to the manufacturer’s instructions. Final DNA concentration and quality were assessed by electrophoresis on [1.5%] agarose gels. After purification, samples were sequenced on the MiSeq platform from Illumina^**®**^, by the *Plateforme d’analyses génomiques* at the *Institut de Biologie Intégrative et des Systèmes* (IBIS) from Université Laval. All proceedings followed the manufacturer’s protocols.

### 16S amplicon sequences analysis

The sequence files are available from the Sequence Read Archive (http://www.ncbi.nlm.nih.gov/sra), BioProject ID: PRJNA385117. The analysis of amplicon sequences was done at the *Institut de Biologie Intégrative et des Systèmes* (IBIS) at *Université Laval*. The demultiplexed, paired-end, fastq sequence files were processed through QIIME^52^. The script *multiple_join_paired_ends.py* was used to join forward and reverse reads. The script *multiple_split_libraries_fastq.py* was used to quality-filter reads at default Phred threshold ≥20. Then, chimeric sequences were identified using the *identify_chimeric_seqs.py* script and removed from all sequences using *filter_fasta.py*. The script *pick_de_novo_otus.py* was used for de novo operational taxonomic unit (OTU) picking. For all downstream taxonomic analyses, the OTU table produced by de novo OTU picking was used, as it does not exclude OTUs absent from online databases (as opposed to closed reference OTU picking). The GreenGenes online database *gg_13_8_otus* was used to annotate OTUs. OTUs with less than 50 reads or that only occurred in one sample were filtered out as a step to improve accuracy and diversity^53^. Samples with a low sequencing depth (<500 reads) were discarded. A rarefaction plot can be found in Supplementary material (Supplementary Figure 1). OTU tables were normalized with the abundance-based metagenomeSeq’s cumulative sum scaling (CSS) transformation^54^ using the QIIME script *normalize_table.py*. The normalized OTU table was used for all downstream analysis.

For all downstream analysis, the fry samples were separated into four developmental stages: before mucus feeding (0–3 DPH), during mucus feeding (4–20 DPH), early after mucus feeding (50–80 DPH), and later after mucus feeding (80–100 DPH). Shannon’s and Chao alpha diversity measures were obtained for each sample with mothur software (*summary.single* command) and were plotted on R version 3.2.1 using package ggplot2^55^. The Yue & Clayton measure of dissimilarity, the Thetayc index^56^, was calculated using mothur (*dist.shared* command). Permutational analyses of variance (PERMANOVA) were performed with the *vegan* package^57^ from R to assess if microbial community composition differences were significant between clusters of samples. The defined clusters were: whole larvae microbiota from 0 to 3 DPH, whole larvae microbiota from 4–20 DPH, fry fecal microbiota from 50 to 80 DPH, fry fecal microbiota from 80 to 100 DPH, adult discus fecal microbiota, discus parent mucus microbiota when feeding fry, discus parent mucus microbiota when not feeding fry and water samples. For each analysis of variance, 10 000 permutations were applied.

The recruitment of bacterial taxa from specific microbial reservoirs, by the fry throughout their development, was assessed using a homemade “recruitment index”. The recruitment index was calculated for each of the four potential microbial reservoirs (environmental water, parent’s skin mucus, parent’s feces, or adult diet). To calculate this recruitment index, we first identified the OTUs that were shared between each microbial reservoir and the fry. After, we calculated the average relative abundance of the total microbial community of each targeted microbial niche, that was represented by the shared OTUs. Finally, we obtained the recruitment index by dividing the relative abundance of the total microbial community represented by shared OTUs in the microbial reservoir by the relative abundance of the total microbial community represented by shared OTUs in the fry gut. When, for a specific microbial reservoir, the recruitment index was >1, it indicated that a high proportion of rare OTUs from this reservoir were positively recruited by the fry gut, where they proliferated and became more ubiquitous. P-values were assessed for the recruitment index by T-tests for two-tailed distribution with unequal variance.

For the three microbial reservoirs that had a significant recruitment index, a network of relative co-abundance between microbial taxa was constructed with Cytoscape version 3.2.1^58^. Only the 50 most abundant OTUs (defined at the lowest taxonomic level possible) were used to construct the network. Spearman’s correlations were calculated between each microbial taxa relative abundance pair using R. A p-value, corrected with Bonferroni, was determined for each Spearman’s correlation value. Significant correlation values used for the network construction had a Spearman’s correlation ≥0.9 and a Bonferroni corrected p-value ≤ 0.0001. The nodes of the network represent a microbial taxa and the edges (i.e. connections between nodes) are attributed to significant correlation between nodes.

Multifactorial Analysis (MFA) were performed (Figs 2g and 4a) with R using the package FactormineR^59^. The MFAs enable multidimensional visualization of the data. They are used to simultaneously study patterns observed in continuous (OTU normalized abundance) and categorical (e.g. parenting versus non-parenting fish) variables. Confidence ellipses were drawn around clusters of data points with default settings of the *plotellipses* function. Clusters with non-overlapping ellipses are considered significantly different (default confidence level of 0.95). Variance equality between clusters of samples (e.g. mucus of parenting fish versus non-breeding fish) was assessed with F-tests on StatPlus v.5.9.92.

## Electronic supplementary material

Supplementary material
