# Supplementary material for: Vertically and horizontally transmitted microbial symbionts shape the gut microbiota ontogenesis of a skin-mucus feeding discus fish progeny
Source: Sci Rep. 2017 Jul 12;7:5263. doi: 10.1038/s41598-017-05662-w (PMC5507859; doi:10.1038/s41598-017-05662-w)
Supplement: Supplementary file 1 — Supplementary material [file 41598_2017_5662_MOESM1_ESM.pdf]

Vertically and horizontally transmitted microbial symbionts shape the gut microbiota ontogenesis of a skin-mucus feeding discus fish progeny

Sylvain, François-Étienne[1]; Derome, Nicolas[1] *

1: Institut de biologie intégrative et des systèmes, Université Laval, Biology Department, 1030, avenue de la Médecine, Quebec (QC), Canada, G1V 0A6
* nicolas.derome@bio.ulaval.ca

Supplementary Information

Figures

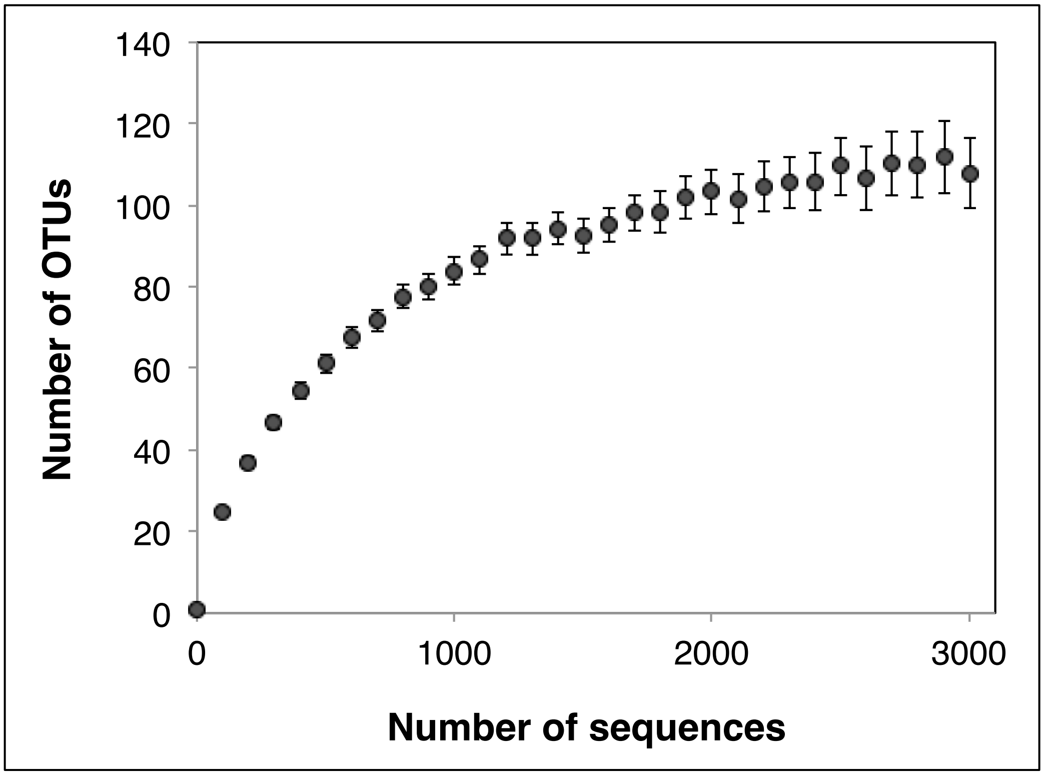

Figure 1: Rarefaction plot constructed with the mean number of OTUs, for every 100 sequences, in all samples. Rarefaction was assessed with mothur (Schloss *et al*. 2009).

Reference

1. Schloss, P. D. *et al*. 2009. Introducing mothur: Open-Source, Platform-Independent, Community-Supported Software for Describing and Comparing Microbial Communities. *Applied and Environmental Microbiology* 75 (23):7537-7541. doi: 10.1128/aem.01541-09.
